# Contribution of multidrug and toxin extrusion protein 1 (MATE1) to renal secretion of trimethylamine-N-oxide (TMAO)

**DOI:** 10.1038/s41598-018-25139-8

**Published:** 2018-04-27

**Authors:** A. Gessner, J. König, M. F. Fromm

**Affiliations:** Institute of Experimental and Clinical Pharmacology and Toxicology, Friedrich-Alexander-Universität Erlangen-Nürnberg, Erlangen, Germany

## Abstract

Trimethylamine-N-oxide (TMAO) gained considerable attention because of its role as a cardiovascular risk biomarker. Organic cation transporter 2 (OCT2) mediates TMAO uptake into renal proximal tubular cells. Here we investigated the potential role of multidrug and toxin extrusion protein 1 (MATE1) for translocation of TMAO across the luminal membrane of proximal tubular cells. HEK293 cells stably expressing OCT2 (HEK-OCT2) or MATE1 (HEK-MATE1) were used for uptake studies. Transcellular transport of TMAO was investigated using monolayers of MDCK control cells (MDCK-Co) as well as single- (MDCK-OCT2, MDCK-MATE1) and double-transfected cells (MDCK-OCT2-MATE1). In line with previous studies, HEK-OCT2 cells revealed a 2.4-fold uptake of TMAO compared to control cells (p < 0.001), whereas no significant uptake was observed in HEK-MATE1. In monolayers of MDCK cells, polarised TMAO transcellular transport was not significantly different between MDCK-Co and MDCK-OCT2 cells, but significantly increased in MDCK-MATE1 (p < 0.05) and MDCK-OCT2-MATE1 cells (p < 0.001). The OCT/MATE inhibitor trimethoprim abolished TMAO translocation in MDCK-OCT2-MATE1 cells (p < 0.05). The present data suggest that MATE1 contributes to renal elimination of TMAO. For selected MATE substrates, such as TMAO, uptake studies using non-polarised MATE-expressing cells can reveal false negative results compared to studies using polarised monolayers.

## Introduction

Cardiovascular disease (CVD) and chronic kidney disease (CKD) are major burdens for public health. During recent years, the small molecule osmolyte trimethylamine-N-oxide (TMAO) was associated with an increased risk for CVD and CKD^1–4^. TMAO appears to be not only a biomarker, but was reported to contribute to the progression of CVD and CKD: it was demonstrated that TMAO inhibits reverse cholesterol transport and promotes foam cell formation^1,5^, enhances platelet responsiveness to agonists^6^, and contributes to renal fibrosis^3^. Particularly interesting is how the endogenous biosynthesis of TMAO may link a typical Western diet rich in choline and L-carnitine to increased CVD risk. The main source of circulating TMAO is the conversion of dietary choline and L-carnitine by intestinal bacteria to trimethylamine (TMA), which is subsequently oxidised by hepatic flavin monooxygenase 3 (FMO3) to TMAO^1,2,5^.

Without undergoing biotransformation^7^ TMAO is almost entirely excreted into the urine by glomerular filtration and with reported renal secretion both in animals and humans^8–10^. Accordingly, TMAO plasma concentrations correlate negatively with kidney function^11^. Transport proteins play a major role for disposition of endogenous compounds and drugs^12–15^. Renal secretion is frequently achieved by transporter-mediated uptake of substrates from blood into renal proximal tubular cells, followed by transporter-mediated export across the apical membrane into urine^16,17^. Recent studies identified organic cation transporter 2 (OCT2, *SLC22A2*) as a transporter mediating uptake of TMAO^9,18^. This transport protein is localised in the basolateral membrane of renal proximal tubular cells and mediates the uptake of organic cations and zwitterions from the blood into cells^19,20^. OCT2 typically works in conjunction with multidrug and toxin extrusion protein 1 (MATE1, *SLC47A1*), which is localised in the apical membrane of renal proximal tubular cells and functions as a proton-coupled antiporter^17,21,22^. OCT2 and MATE1 have a large overlap in substrate spectrum, in which uptake by OCT2 and secretion by MATE1 takes place in a coordinated manner^12,13,17,22–24^. However, surprisingly neither Teft *et al*. nor Miyake *et al*. could characterise the OCT2 substrate TMAO as a substrate of human MATE1 or of the mouse ortholog Mate1, respectively^9,18^.

The physiological role of MATE1-mediated transport is assumed to export substrates out of cells in exchange for a proton^17,21,22^. Nevertheless, MATE1 function is frequently studied in uptake experiments, in which a proton gradient out of the cells is created, for example by extracellular alkalinisation^25–27^. Accordingly, the experiments by Teft *et al*.^18^ and Miyake *et al*.^9^ were both investigating transport of TMAO by MATE1 in the direction of uptake.

Monolayers of double-transfected OCT2/MATE1 cells constitute a very useful model to study polarised basal to apical translocation and allow investigation of the importance of MATE1-mediated export^27–30^. Therefore, the aim of this study was to investigate the role of MATE1 in conjunction with OCT2 in the transcellular transport of TMAO using a polarised cell system stably expressing OCT2 and MATE1 simultaneously, which reflects the anticipated role of both transporter proteins in the kidney.

## Results

### Intracellular accumulation of TMAO in HEK-OCT2 and HEK-MATE1 cells

**Figure 1:**
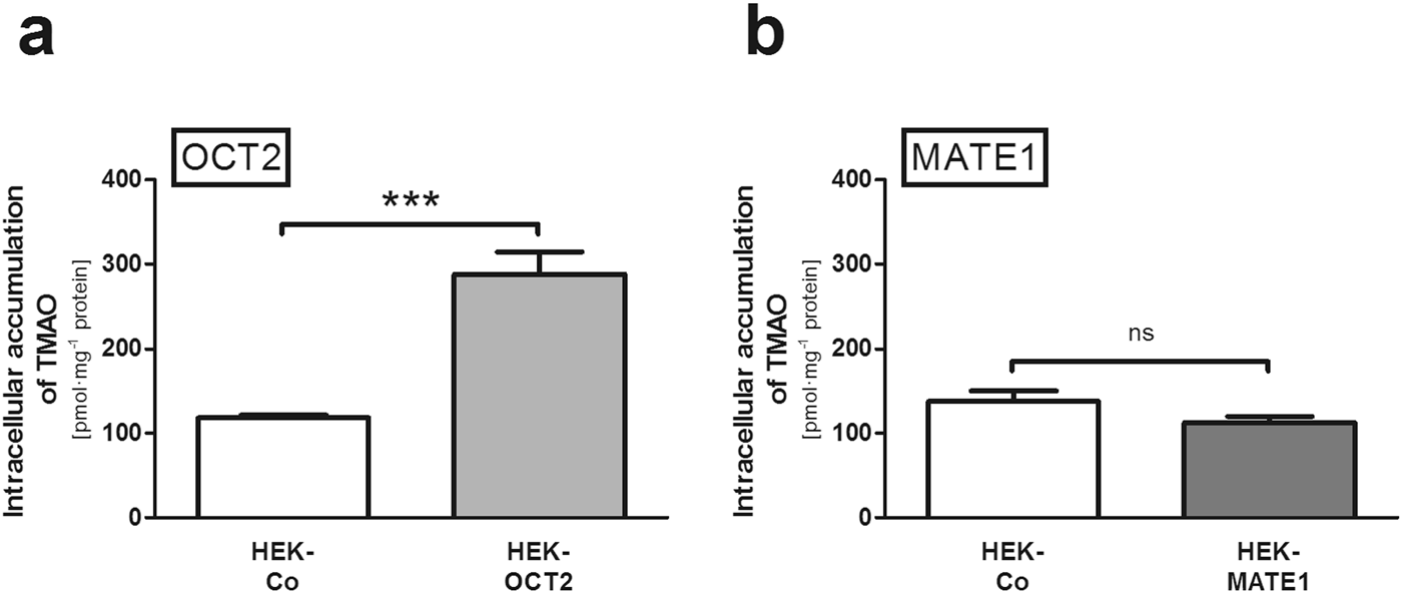
Intracellular accumulation of TMAO (10 µM) by HEK cells expressing OCT2 (HEK-OCT2) (**a**) or MATE1 (HEK-MATE1) (**b**) and the respective control cells (HEK-Co) after 2 min. Data points are 4 × n = 2 for OCT2 and 2 × n = 2 for MATE1, mean ± SEM, ***p < 0.001 vs. HEK-Co.

TMAO accumulation in HEK-OCT2 cells was significantly higher than in control cells (2.4-fold; p < 0.001; Fig. 1a), in line with the results by Teft *et al*.^18^ and Miyake *et al*.^9^. As expected, intracellular accumulation of the model substrate MPP^+^, serving as a positive control, was increased 12.9-fold in HEK-OCT2 cells and 2.8-fold in HEK-MATE1 cells, respectively, compared to accumulation in HEK-Co cells (p < 0.001, data not shown). In contrast, in HEK-MATE1 cells intracellular accumulation of TMAO, analysed under the same experimental conditions, was not statistically different from intracellular accumulation in the respective HEK-Co control cells (Fig. 1b), which is again in line with the findings of Teft *et al*.^18^ and Miyake *et al*.^9^.

### Transcellular transport of TMAO in monolayers of MDCK cells

**Figure 2:**
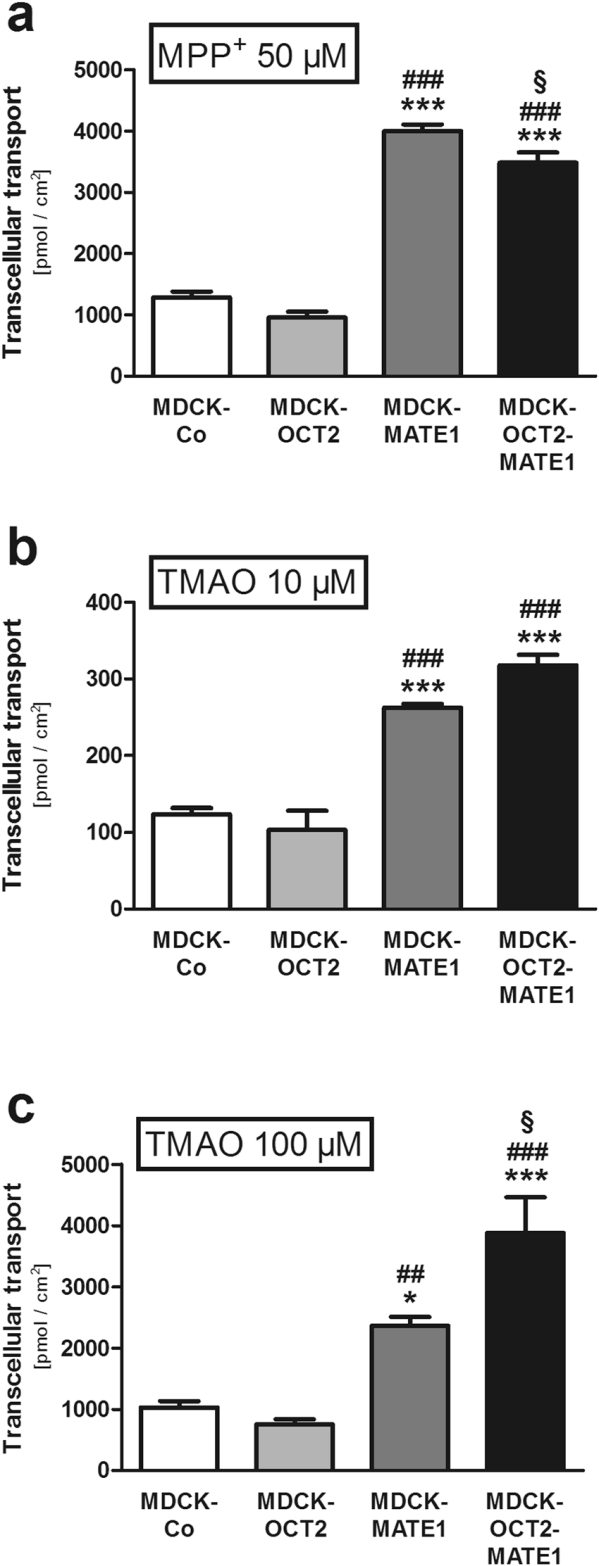
Transcellular transport of MPP^+^ and TMAO [MPP^+^ (50 µM) (**a**), TMAO (10 µM) (**b**), and TMAO (100 µM) (**c**)] across polarised grown monolayers of MDCK cells expressing OCT2 (MDCK-OCT2), MATE1 (MDCK-MATE1), or both OCT2 and MATE1 (MDCK-OCT2-MATE1) and control cells (MDCK-Co) after 60 min. Each data point is 2 × n = 3, mean ± SEM, *p < 0.05 vs. MDCK-Co, ***p < 0.001 vs. MDCK-Co, ^##^p < 0.01 vs. MDCK-OCT2, ^###^p < 0.001 vs. MDCK-OCT2, ^§^p < 0.05 vs. MDCK-MATE1.

**Figure 3:**
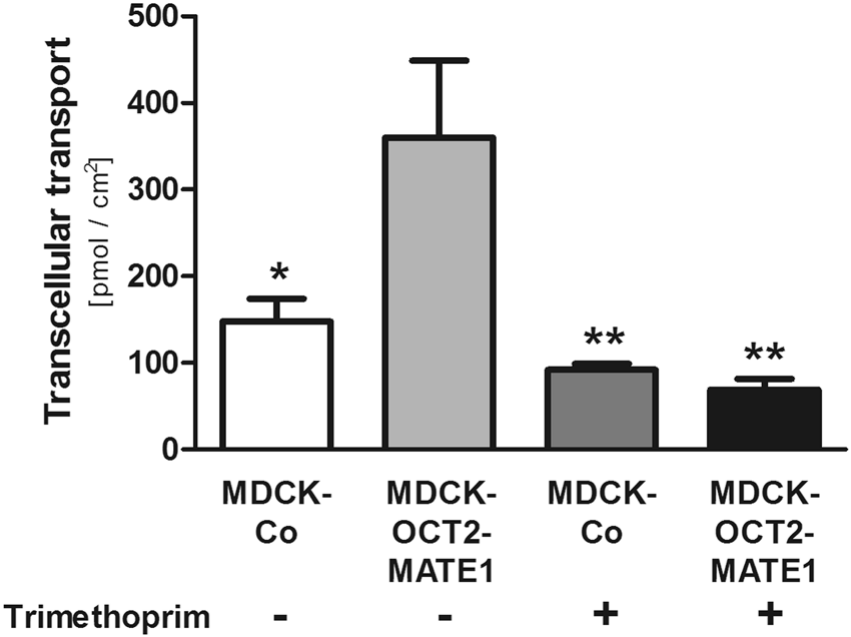
Transcellular transport of TMAO (10 µM) across polarised grown monolayers of MDCK cells expressing both OCT2 and MATE1 (MDCK-OCT2-MATE1) and control cells (MDCK-Co) after 60 min without (−) or with (+) addition of trimethoprim (100 µM) to the basal compartment. Each data point is 2 × n = 2, mean ± SEM, *p < 0.05 vs. MDCK-OCT2-MATE1 without trimethoprim, **p < 0.01 vs. MDCK-OCT2-MATE1 without trimethoprim.

Transcellular TMAO transport from the basal to the apical compartment was consistently higher in double-transfected MDCK-OCT2-MATE1 and single-transfected MDCK-MATE1 cells compared to MDCK-Co or single-transfected MDCK-OCT2 cells (Fig. 2). At a TMAO concentration of 10 µM transcellular transport in MDCK-OCT2-MATE1 cells was increased 2.6-fold and 3.1-fold vs. MDCK-Co and MDCK-OCT2 cells, respectively (p < 0.001 each; Fig. 2b). Compared to MDCK-MATE1 cells there was no significant difference detected (Fig. 2b). At a TMAO concentration of 100 µM transcellular transport in MDCK-OCT2-MATE1 cells was increased 3.8-fold and 5.2-fold vs. MDCK-Co and MDCK-OCT2 cells, respectively (p < 0.001 each; Fig. 2c), and 1.6-fold vs. MDCK-MATE1 cells (p < 0.05; Fig. 2c). No significant difference in transcellular transport was observed between MDCK-OCT2 and MDCK-Co cells. For MDCK-MATE1 cells, the transcellular TMAO transport was significantly more pronounced than in MDCK-Co or MDCK-OCT2 cells. At a TMAO concentration of 10 µM it was increased 2.1-fold vs. MDCK-Co and 2.5-fold vs. MDCK-OCT2 cells (p < 0.001 each; Fig. 2b). The increase at a TMAO concentration of 100 µM was 2.3-fold vs. MDCK-Co cells (p < 0.05; Fig. 2c) and 3.2-fold vs. MDCK-OCT2 cells (p < 0.01; Fig. 2c). Transcellular transport of the prototypical OCT/MATE substrate MPP^+^, serving as a positive control (Fig. 2a), was in line with previous studies^28^. In MDCK-OCT2-MATE1 cells, addition of the OCT/MATE inhibitor trimethoprim significantly reduced vectorial transport of TMAO to the apical compartment to the level of control cells (p < 0.01; Fig. 3).

### Intracellular accumulation of TMAO in monolayers of MDCK cells

**Figure 4:**
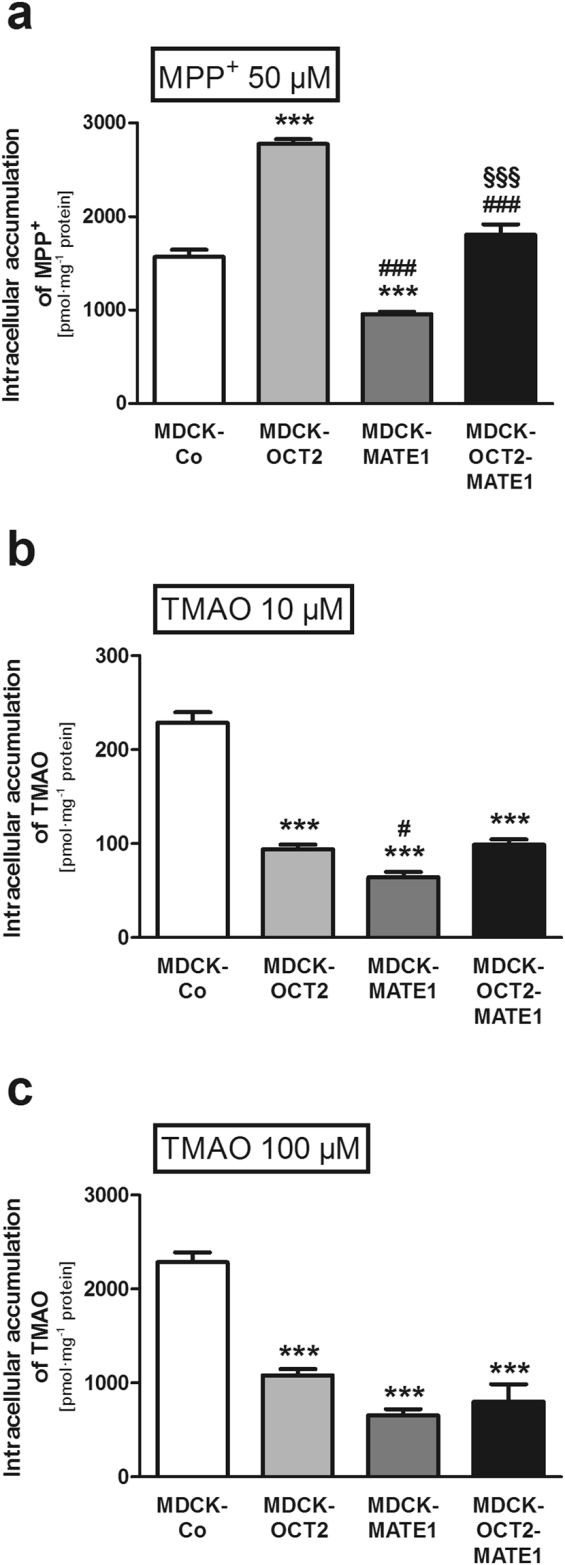
Intracellular accumulation of MPP^+^ and TMAO [MPP^+^ (50 µM) (**a**), TMAO (10 µM) (**b**), and TMAO (100 µM) (**c**)] in MDCK cells grown as polarised monolayers expressing OCT2 (MDCK-OCT2), MATE1 (MDCK-MATE1), or both OCT2 and MATE1 (MDCK-OCT2-MATE1) and control cells (MDCK-Co) after 60 min. Each data point is 2 × n = 3, mean ± SEM, ***p < 0.001 vs. MDCK-Co, ^#^p < 0.05 vs. MDCK-OCT2, ^###^p < 0.001 vs. MDCK-OCT2, ^§§§^p < 0.001 vs. MDCK-MATE1.

**Figure 5:**
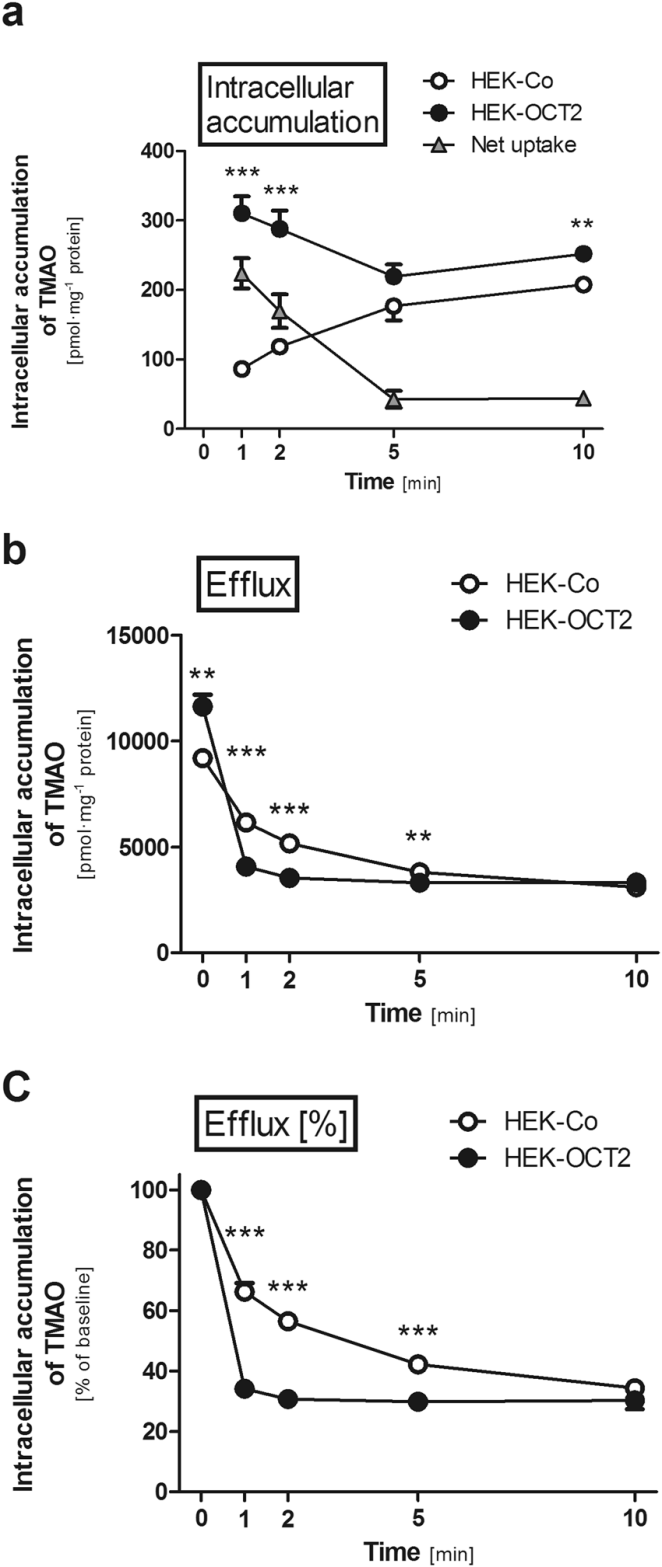
Time-dependent intracellular accumulation and efflux of TMAO (10 µM) in HEK cells expressing OCT2 (HEK-OCT2) and control cells (HEK-Co). (**a**) TMAO intracellular accumulation measured at 1, 2, 5, and 10 min. (**b**) Efflux experiments: cells were preincubated for 60 min with TMAO (300 µM) and subsequently the extracellular medium was replaced with buffer without TMAO. Intracellular accumulation of TMAO was measured after 0 (baseline), 1, 2, 5, and 10 min. (**c**) The same data as in (b) with intracellular accumulation at baseline set to 100%. Data points are 2 × n = 2 at 1, 5, and 10 min and 4 × n = 2 at 0 and 2 min, mean ± SEM, **p < 0.01 vs. HEK-Co, ***p < 0.001 vs. HEK-Co.

Using the same experimental conditions, intracellular TMAO accumulation was at least 2.1-fold reduced in all cell types investigated in comparison to MDCK-Co cells for both concentrations tested (p < 0.001 each; Fig. 4b,c). With respect to OCT2, this result appears to be in contrast to the intracellular accumulation observed in HEK-OCT2 cells after 2 min (Fig. 1a), deeming it necessary to further investigate the time dependent accumulation of TMAO in single-transfected cells expressing OCT2 (see below and Fig. 5). The control experiments with MPP^+^ revealed the expected results for both OCT2 and MATE1 (Fig. 4a) and are in line with previous studies^28^.

### Time dependent intracellular accumulation and efflux of TMAO in HEK cells

TMAO accumulation in HEK-OCT2 cells was significantly higher at 1 and 2 min than in HEK-Co cells (3.6 and 2.4-fold respectively; p < 0.001 each; Fig. 5a). At 5 min no significant difference could be detected between HEK-Co and HEK-OCT2 cells. Interestingly, for HEK-OCT2 cells the intracellular TMAO levels detected at 5 and 10 min were lower than those at 1 and 2 min, while for HEK-Co cells intracellular levels continually increased over time, largely diminishing net uptake after 5 and 10 min (Fig. 5a). These results indicate an OCT2-mediated efflux of TMAO, which was further investigated by pre-loading experiments.

After a loading phase of 60 min with TMAO (300 µM) the intracellular levels detected in HEK-OCT2 cells were significantly higher than in HEK-Co cells (1.3-fold; p < 0.01; Fig. 5b). After removing extracellular TMAO, intracellular levels of TMAO in HEK-OCT2 cells rapidly dropped to 34% of the initial concentration within 1 min and after 2 min a constant concentration of ~30% of the initial concentration at all time points investigated was reached. Intracellular concentration of TMAO in control cells gradually decreased over time and reached a level of 34% of the initial concentration after 10 min (Fig. 5c).

## Discussion

The aim of this study was to investigate MATE1-mediated transport of TMAO across cell monolayers in conjunction with OCT2. The results of our experiments with polarised monolayers of MDCK cells demonstrate that TMAO is a substrate of MATE1, because (1) basal to apical TMAO translocation was considerably higher in the MDCK-OCT2-MATE1 and MDCK-MATE1 monolayers compared to the other cell lines, (2) the OCT/MATE inhibitor trimethoprim^30,31^ abolished polarised transport in the double-transfected cells and (3) both MATE1 expressing MDCK cell lines accumulated significantly less TMAO compared to the control cells in the monolayer experiments. The identification of TMAO as a risk factor in CVD and CKD, being associated with their development and their outcome, has raised considerable attention to factors that account for interindividual variations of its plasma concentration. Some of these are a diet rich in precursors of TMAO, composition of the gut microbiome, kidney function, age, and co-morbidities^1–5,32,33^. As renal excretion of non-metabolised TMAO accounts for more than 90% of its elimination^7^, it is mandatory to understand the molecular mechanisms of renal elimination of TMAO.

It is generally believed that TMAO is actively secreted in the kidneys^3,18^ in addition to glomerular filtration. Experiments from animal studies point to a contribution of transporter-mediated renal secretion to overall TMAO elimination^8,9,18^. Miyake *et al*. found a considerably higher renal clearance of TMAO in wild-type mice compared to Oct1/Oct2-double knock-out mice, but they report in the discussion (methods and data are not shown) that the preferential Mate inhibitor pyrimethamine surprisingly did not affect renal clearance of TMAO^9^. The reason for this observation is unclear, but according to our data we would have expected a reduction of TMAO renal clearance by appropriate dosages/concentrations of pyrimethamine. In humans, one study reported for healthy subjects a mean renal TMAO clearance of 218 ml/min based on a 24 h collection of urine and one single blood sample^10^. Due to the pronounced diurnal variation of TMAO plasma concentrations^9,34^, the time point of blood sampling could affect the determined TMAO renal clearance. Miyake *et al*. reported a mean TMAO renal clearance in young healthy subjects using data from an eight hour time interval of 148 ml/min^9^. Surprisingly, creatinine clearance was almost identical in these subjects (146 ml/min). In our opinion, further clinical studies in healthy volunteers are necessary to define the extent of TMAO renal secretion with a particular focus on diurnal variations of TMAO plasma concentrations and standardisation of diet.

Two groups independently identified human OCT2 as a low affinity TMAO uptake transporter (determined K_m_ values of 73.67 mM and 7.37 mM, respectively) from blood into proximal tubular cells^9,18^. Surprisingly, in neither of those studies MATE1-/Mate1-mediated transport was detectable, though MATE1/Mate1 has a large overlap with OCT2 regarding substrate specificity^12,13,17,22–24^. Our initial studies using single-transfected HEK-MATE1 cells confirmed these findings by Teft *et al*.^18^ and Miyake *et al*.^9^ (Fig. 1). However, in all of these studies the experimental setup tests a MATE1-/Mate1-mediated uptake into the cells by an outward oriented proton gradient. This does not reflect the assumed function of MATE1 in the kidney, being located in the brush border membrane and mediating the efflux of substrates from the intracellular compartment into urine^17,21,22^. Polarised grown monolayers of MDCK cells may represent the physiological situation better than unpolarised grown HEK cells. Our results from experiments using such cells stably expressing either OCT2 or MATE1 or both OCT2 and MATE1 clearly indicate a contribution of MATE1 to the transcellular transport of TMAO (Figs 2 and 3).

While the direction of transport by the proton-coupled cation exchanger MATE1 largely depends on the pH gradient between extra- and intracellular medium^17,21,22^, substrate affinity on the extra- and intracellular moieties of the transporter protein may be different^35^. A high affinity on the intracellular moiety along with a comparatively low affinity on the extracellular would result in higher rate of efflux, independently of the direction of the pH gradient. The present data suggest that this might be the case for TMAO. An efficient transport of TMAO across membranes by MATE1 is only detectable if TMAO binds to the intracellular moiety of MATE1 as it was possible in the MDCK monolayer studies, but not in the above mentioned uptake experiments using HEK cells. This implies that for certain substrates, such as TMAO, uptake studies with non-polarised cells could reveal false negative results compared to efflux studies using polarised monolayers. This is potentially important, because recently the FDA issued a draft guideline for testing *in vitro* transporter-mediated drug-drug interactions for investigational drug products, in which non-polarised cells (such as HEK cells) are mentioned as one adequate cell system to test for interactions via MATE transporters^36^. In line with the present findings, the antiretroviral drug lamivudine showed only modest, yet significant, uptake in HEK-MATE1 cells, but a pronounced, highly significant transcellular transport in MDCK cells expressing MATE1^27^.

Other transporters might contribute to TMAO renal secretion via the luminal membrane of proximal tubular cells. Teft *et al*. showed, using double-transfections of HeLa cells with OCT2 and the respective efflux transporter that TMAO seems to be a substrate for other export proteins expressed in the kidney such as P-glycoprotein, BCRP, and MRP2^18^. To the best of our knowledge it has not yet been studied whether TMAO is also a substrate of renally expressed MATE2-K. On the background of increasing polymedication, especially in the elderly, the inhibition of one or several transport proteins by drugs may account for a rise in TMAO levels in plasma or intracellularly. Some studies suggest that about 40% of all prescribed drugs are positively charged at physiological pH, providing various chances of interactions via OCT or MATE transporters^37^. Consequences of such interactions on TMAO plasma concentrations or renal clearance are yet to be elucidated. It should also be considered that co-administration of OCT/MATE inhibitors may be a confounder in epidemiological studies using TMAO as a biomarker.

Time dependent accumulation in HEK-OCT2 cells demonstrated, that there initially is a rapid increase of intracellular TMAO within 1 min, while at later time points a lower and somewhat constant level is reached (Fig. 5). In HEK-Co cells the intracellular accumulation of TMAO constantly increased at each time point investigated, resulting in a largely diminished net uptake when comparing the intracellular accumulation in HEK-OCT2 and HEK-Co cells after 5 and 10 min (Fig. 5a). Our data indicate that OCT2 functions not only as an uptake, but also as an efflux transporter for TMAO. After pre-loading with TMAO at a concentration of 300 µM, the efflux from HEK-OCT2 cells occurs rapidly within 1 min, and reaches equilibrium after 2 min (Fig. 5b,c). Efflux capacities of OCT2 have already previously been suggested^19,20,37,38^. Under physiological conditions, OCT2-mediated efflux may be a protective mechanism against high intracellular concentrations. In combination with other uptake and efflux proteins OCT2 could significantly contribute to keeping a constant intracellular level of TMAO. A disturbance of this constant level might lead to TMAO rising to noxious levels in the cells. A yet to be determined intracellular concentration may be beneficial for kidney cells to counteract osmotic stress, similar to the function of TMAO in some marine animals^39^, while unphysiologically high concentrations may enhance the formation of non-functional protein aggregates^40,41^. The rapid transport of TMAO across membranes in both directions by OCT2 could account for diurnal variations of TMAO plasma levels in response to dietary intake of precursors or TMAO itself^9,34^, creating a need to quickly adapt intracellular levels.

In conclusion, our data demonstrate that MATE1 transports TMAO across cell membranes in conjunction with OCT2 and thereby may contribute to its renal secretion. In addition, we have shown that OCT2 does not only mediate the influx of TMAO, but also its efflux from cells. Furthermore, there is an indication that for some substrates MATE/Mate uptake studies in non-polarised cells, such as HEK, may lead to false negative results, as this setup does not adequately reflect the anticipated physiological role of these transport proteins. In these instances, efflux studies using polarised grown cell monolayers might resemble a more suitable model of the physiological situation.

## Methods

### Chemicals

Unlabelled trimethylamine-N-oxide (TMAO), 1-methyl-4-phenylpyridinium (MPP^+^), and trimethoprim were obtained from Sigma-Aldrich (Taufkirchen, Germany). [^3^H]TMAO (60 Ci/mmol) and [^3^H]MPP^+^ (80 Ci/mmol) were obtained from Biotrend Chemikalien GmbH (Cologne, Germany). Poly-D-lysine hydrobromide (PDL) and sodium butyrate were purchased from Sigma-Aldrich (Taufkirchen, Germany). Cellstar 12-well cell culture plates and ThinCert^TM^-TC Inserts (12 well, pore size 0.4 µm, translucent, filter area 1.1 cm^2^) were from Greiner Bio-One GmbH (Frickenhausen, Germany) or Sarstedt AG & Co. KG (Nümbrecht, Germany). Cell culture media supplements were obtained from Thermo Fisher Life Technologies GmbH (Darmstadt, Germany). All other chemicals and reagents, unless stated otherwise, were obtained from Carl Roth GmbH + Co. KG (Karlsruhe, Germany).

### Cell culture

Generation, characterisation, and cell culture conditions of the cell lines have been described before^28,30,42,43^. In brief, assays were performed using [^3^H]-labelled substrate and single- and double-transfected human embryonic kidney 293 (HEK) or Madin-Darby canine kidney II (MDCK) cells transfected with the empty vector only (HEK-Co or MDCK-Co), or expressing human OCT2 (HEK-OCT2 or MDCK-OCT2), human MATE1 (HEK-MATE1 or MDCK-MATE1), or human OCT2 together with human MATE1 (MDCK-OCT2-MATE1). Cells were cultured in minimal essential medium containing 10% heat-inactivated fetal bovine serum, 100 U/l penicillin, 100 µg/ml streptomycin, and either G418 (800 µg/ml) or hygromycin B (260 µg/ml) at 37 °C and 5% CO_2_ with subculture as required. Trypsin (0.05%)-EDTA (0.02%) solution was used to detach cells. All cell culture media supplements were obtained from Thermo Fisher Life Technologies GmbH (Darmstadt, Germany).

HEK cells were used for investigating cellular uptake and efflux, while monolayers of MDCK cells were used to study transcellular transport from the basal to the apical compartment and intracellular accumulation of the compounds. For all experiments the well characterised OCT2/MATE1-substrate MPP^+^ served as a positive control.

### Uptake transport assays with HEK cells

Cellular uptake assays were performed as previously described^30^ with minor modifications. In brief, cells were seeded in PDL-coated (0.1 mg/ml) 12-well plates at a density of 7 × 10^5^ cells per well. 24 h after seeding, cells were induced with 10 mM sodium butyrate to enhance protein expression^44^. 48 h after seeding, transport experiments were performed.

Cells were washed once with incubation medium at 37 °C. Afterwards, cells were incubated with medium containing a mixture of unlabelled TMAO and [^3^H]TMAO at 37 °C for 1, 2, 5, or 10 min. The incubation medium for the experiments contained 142 mM NaCl, 5 mM KCl, 1 mM K_2_HPO_4_, 1.2 mM MgSO_4_, 1.5 mM CaCl_2_, 5 mM glucose, and 12.5 mM HEPES. The pH value of the incubation medium for the washing step as well as of the medium containing radioactively labelled substrate was 7.3 for HEK-OCT2 cells and the respective vector controls and 8.0 for HEK-MATE1 cells and the respective vector controls. At the end of the incubation period, the medium was removed, and cells were rinsed thrice with ice-cold incubation medium (pH 7.3 or pH 8.0). Then, cells were lysed with sodium dodecyl sulfate (SDS, 0.2%). The amount of radioactivity in the cell lysate was measured using liquid scintillation counting (Tricarb 2800; Perkin Elmer Life and Analytical Sciences Inc., Rodgau-Jugesheim, Germany), and protein concentrations were measured by bicinchoninic acid assay (Pierce BCA protein assay kit, Thermo Fisher Life Technologies GmbH (Darmstadt, Germany)).

### Efflux transport assays with HEK cells

Efflux transport assays were performed as previously described^45^ with minor modifications. Cell culture conditions and seeding of cells was performed in the same way as described for the uptake experiments.

Cells were washed once with incubation medium at 37 °C. Afterwards, cells were preloaded with incubation medium of the same composition as in the uptake experiments containing a mixture of unlabelled TMAO and [^3^H]-labelled TMAO, at a total substrate concentration of 300 µM at 37 °C for 60 min. At the end of the incubation period, the medium was removed, and cells were rinsed once with prewarmed incubation medium (pH 7.3). Then, cells were incubated with efflux buffer at pH 7.3 for HEK-OCT2 cells and the respective vector controls at 37 °C. Immediately after washing (0 min; baseline) and after 1, 2, 5, and 10 min cells were put on ice to stop transport. After the removal of the supernatant, cells were rinsed thrice with ice-cold incubation medium (pH 7.3). Then, cells were lysed with SDS 0.2%. The concentration of radioactivity in the cell lysate, as well as the protein concentration, was measured as for the uptake experiments.

### Transcellular transport and cellular uptake assays with MDCK cells

The intracellular accumulation and transcellular transport experiments of TMAO or MPP^+^ were performed as previously described in monolayers of single- and double-transfected MDCK cells^28^. In brief, cell monolayers were grown in cell culture inserts on porous membranes. For all experiments, 5 × 10^5^ cells per well were used. Cells were grown to confluence for 72 h, induced with 10 mM sodium butyrate 48 h after seeding for 24 h, and subsequently used for transport experiments.

In general, after the culture medium was removed from both sides of the monolayers, cells were washed with incubation medium of the same composition as described in the previous sections at 37 °C. Experiments were started by replacing the medium in the basal compartment with medium containing a mixture of unlabelled TMAO or MPP^+^ and [^3^H]-labelled TMAO or [^3^H]MPP^+^, respectively. For investigating inhibition by trimethoprim, the inhibitor was added at a concentration of 100 µM to the incubation medium in the basal compartment containing unlabelled and radiolabelled substrate for the whole incubation period. The pH of the medium on the basal side was 7.3, the pH on the apical side was 6.5. Cells were incubated at 37 °C, and aliquots of 400 µl were taken after 60 min. To measure the cellular accumulation of the labelled test compounds, the medium was removed at the end of the incubation period and the cell monolayers were rapidly rinsed three times with ice-cold incubation medium (pH 7.3). Filters were detached from the chambers and cells were lysed with SDS 0.2%. The radioactivity of the collected apical medium and the solubilised cell monolayers and protein concentrations of the cell lysates was measured in the same way as for the experiments with HEK cells.

### Statistical analysis

Each concentration and time point was investigated on two to four separate days with two or three wells per day (i.e. n = 4 to n = 8). All data are presented as mean ± standard error of the mean (SEM). Substrate uptake by HEK and MDCK cells was normalised with respect to protein concentrations of the cell lysate. Transcellular transport of substrate across monolayers of MDCK cells was normalised with respect to the surface of the transwell filters. Net transport data was calculated as the difference of uptake into cells transfected with the respective transporter and uptake into the corresponding empty vector control cells. Pairwise comparisons were analysed for statistical significance with two-tailed unpaired Student’s *t*-test and multiple comparisons by one-way ANOVA with subsequent Tukey-Kramer multiple comparison test by using GraphPad Prism 5.01 (GraphPad Software, San Diego, CA, USA). A value of p < 0.05 was considered as statistically significant.

### Availability of Materials and Data

No restrictions to availability of data in this manuscript is made. Any information can be obtained by contacting the authors.
